# Identification and characterization of murine glycoprotein 2‐expressing intestinal dendritic cells

**DOI:** 10.1111/sji.13219

**Published:** 2022-10-17

**Authors:** Katarzyna M. Luda, Clement Da Silva, Fatemeh Ahmadi, Allan Mcl. Mowat, Hiroshi Ohno, Knut Kotarsky, William W. Agace

**Affiliations:** ^1^ Immunology Section, Department of Experimental Medical Science Lund University Lund Sweden; ^2^ Centre for Immunobiology, Institute of Infection, Immunity and Inflammation, College of Medicine, Veterinary Medicine and Life Sciences University of Glasgow Glasgow UK; ^3^ RIKEN Center for Integrative Medical Sciences Yokohama Japan; ^4^ Mucosal Immunology Laboratory, Department of Health Technology Technical University of Denmark Lyngby Denmark

**Keywords:** cDC2, dendritic cell, homeostasis, intestine, IRF4, retinoic acid, TGFβR1

## Abstract

The intestinal lamina propria (LP) contains distinct subsets of classical dendritic cells (cDC), each playing key non‐redundant roles in intestinal immune homeostasis. Here, we show that glycoprotein 2 (GP2), a GPI‐anchored protein and receptor for bacterial type‐I fimbriae, is selectively expressed by CD103^+^CD11b^+^ cDC in the murine small intestine (SI). GP2 expression was induced on CD103^+^CD11b^+^ cDC within the SI‐LP and was regulated by IRF4, TGFβR1‐ and retinoic acid signalling. Mice selectively lacking *Gp2* on CD103^+^CD11b^+^ cDC (*huLang‐Cre*.*gp2* ^*fl/fl*^ mice) had normal numbers and proportions of innate and adaptive immune cells in the SI‐LP suggesting that GP2 expression by CD103^+^CD11b^+^ cDC is not required for intestinal immune homoeostasis.

## 1 INTRODUCTION

Classical dendritic cells (cDC) are the essential antigen‐presenting cells of the body important for the generation of tolerogenic, immunogenic and pathogenic adaptive immune responses. Within the intestine, cDC are found diffusely distributed within the intestinal lamina propria (LP). They are also present within gut‐associated lymphoid tissues (GALT), including the Peyer's patches and isolated lymphoid follicles. Within the LP, cDC scan the tissue for foreign and self‐antigen and subsequently migrate via lymphatics to intestinal‐draining mesenteric lymph nodes (MLN) where they present antigen to T cells. The context of this interaction is essential in determining the outcome of the ensuing adaptive immune response.

Murine cDC consist of two major cDC subsets; cDC1 and cDC2 that each play non‐redundant roles in intestinal immune homeostasis. Intestinal cDC1, identified by their expression of XCR1, or as CD103^+^CD11b^−^ cDC, cross‐present epithelial derived antigen to CD8^+^ T cells and are critical for cross‐tolerance as well as intestinal Th1 and intra‐epithelial lymphocyte (IEL) homeostasis.^1^, ^2^, ^3^, ^4^ In contrast, intestinal cDC2, that include CD103^+^CD11b^+^ and CD103^−^CD11b^+^ cDC, are essential for intestinal Th2 responses, and intestinal Th17 homeostasis.^5^, ^6^, ^7^, ^8^, ^9^, ^10^, ^11^ The phenotype and function of intestinal cDC is also regulated by local environmental signals. For example, murine small intestinal (SI) LP (SI‐LP) cDC1 and cDC2 are locally imprinted with the ability to generate the Vitamin A metabolite, retinoic acid (RA), endowing these cells with an enhanced ability to generate CCR9^+^α4β7^+^ gut tropic T cells.^5^, ^10^, ^12^, ^13^, ^14^, ^15^, ^16^ Furthermore, local TGFβ signalling modulates the phenotype and abundance of murine SI‐LP cDC2 subsets.^17^ The extent to which local signals imprint phenotypic and functional specificities in intestinal cDC remains unclear.

Glycoprotein 2 (GP2) is a 78 kDa glycosylphosphatidylinositol (GPI)‐anchored membrane protein expressed by several epithelial glandular tissues, acinar cells of the pancreas and microfold (M‐) cells in the follicular‐associated epithelium (FAE) of Peyer's patches (PP).^18^, ^19^, ^20^, ^21^ Functionally, GP2 binds, in a mannose‐dependent manner, to the FimH adhesin of type‐I‐fimbriated bacteria and its expression by M cells promotes the uptake of and adaptive immune responses to these bacteria.^22^, ^23^, ^24^ Furthermore, pancreas derived‐GP2 was recently suggested to reduce the adhesion and penetration of pathogenic *Escherichia coli* into the intestinal mucosa and to be protective in DSS‐induced colitis.^25^ In addition to its ability to bind type 1 fimbriated bacteria, GP2 has also been proposed to have immunomodulatory functions on innate and adaptive immune cells.^26^ Finally, GP2 is one of the major targets of autoantibodies in Crohn's disease.^26^, ^27^, ^28^, ^29^, ^30^, ^31^

Transcriptional profiling of murine intestinal cDC has suggested that *Gp2* transcripts are enriched in SI‐LP CD103^+^CD11b^+^ cDC.^17^ Here, we demonstrate that GP2 is selectively expressed by a large proportion of murine SI‐LP CD103^+^CD11b^+^ cDC2 and their migratory equivalents in draining MLN, while being largely absent from other immune cells in the intestine. GP2 expression on CD103^+^CD11b^+^ cDC2 was regulated by IRF4 and by environmental cues, including both TGFβ and retinoic acid. Finally, we found that mice whose CD103^+^CD11b^+^ cDC2 lack *gp2* have normal numbers and proportions of innate and adaptive immune cells in the SI‐LP, indicating that its expression by cDC2 is not required for intestinal immune homeostasis.

## 2 MATERIALS AND METHODS

### 2.1 Mice

The following mouse strains were used in the course of this study: *Itgax‐Cre*,^32^ C57BL/6, C57BL/6 (CD45.1), C57BL/6 (CD45.1/CD45.2), B6.129S1‐*Irf4* ^tm1Rdf^/J,^33^ *dnRAR* ^lsl/lsl,^ ^34^ *human‐Langerin‐Cre* (*huLang‐Cre*, kindly provided by Dr D. Kaplan, University of Minnesota, Minneapolis, USA),^35^ *MyD88* ^flox^ ^36^ and *Gp2* ^flox^ mice.^25^ All mice were bred and maintained at the Biomedical Center (BMC), Lund University or Clinical Research Center, Malmö. Animal experiments were performed in accordance with ethical permission from the Lund/Malmö Animal Ethics Committee.

### 2.2 Mouse genotyping

Genotyping was performed on mouse ear biopsies using the following oligonucleotides: GP2 KO, flox and wt mice; GP2_FW 5′ CATCAACAAAACGGGACTCATA3′, GP2_RV1 5′ CCTAGAAGGGACTACACTGG3′, GP2_RV2 5′ TTTTTAAAGGAATGAAAGGCTGT3′. hu‐Langerin Cre mice; iCRE_FW 5′ GCACCTGGGCCAGCTCAACAT3′, iCRE_RV 5′ TGGTCAAAGTCAGTGCGTTCA3′. IRF4 flox and wt mice; IRF4_FW 5′ CCACTCTCTGCTTCCCTGTC3′ and IRF4_RV 5′ CTCTCGACCAATTCCTCAAAGT3′. To discriminate IRF4 flox and delta mice; IRF4_FW1 5′ AGCTTGCCGTAGGTGGCATCG3′, IRF4_FW2 5′ CCACTCTCTGCTTCCCTGTC3′ and IRF4_RV 5′ CTTCCTCATCTCCGGGCCTTTCG3′. RARdn flox and wt mice; ROSA1 (common) 5′ AAAGTCGCTCTGAGTTGTTAT3′, ROSA2 5′ GCGAAGAGTTTGTCCTCAACC3′ and ROSA3 (wt) 5′ GGAGCGGGAGAAATGGATATG3′.

### 2.3 Cell isolation

For isolation of LP cells, single‐cell suspensions were generated as previously described.^15^ In some instances (see Section 3), Liberase TM (0.3 WuenschU/mL, Roche) was replaced with Collagenase II (230 U/mL, Gibco). For isolation of cDC from non‐digested intestine and lung, tissues were cut into small pieces, placed on a petri‐dish filled with R10 medium (RPMI 1640 containing with 10% FCS (Sigma‐Aldrich), 1 mM sodium pyruvate (Gibco), 10 mM HEPES, 100 U/mL penicillin and 100 μg/mL streptomycin, 50 μg/mL gentamycin (Gibco), 50 μM 2‐mercaptoethanol (Gibco)) at 37°C, 5% CO_2_, and cells collected from the culture after 20 hours. For isolation of MLN and splenic cells, organs were mashed through a 70 μm cell strainer, except in Figure 3D where MLN were digested with collagenase II (230 U/mL) and DNAse I (30 μg/mL, Roche), as described previously.^15^ Red blood cells in splenic cell suspensions were lysed using ACK lysing buffer.

### 2.4 Antibodies and flow cytometry

The following antibodies were used for flow cytometry: NK1.1 (PK 136), CD19 (6D5 or 1D3), CD3e (17A2 or 145‐2C11), MHC II(IA/I‐E) (M5/114.15.2), CD11b (M1/70), CD8α (53‐6.7), Ly6C (HK1.4 or AL‐21), CD45.2 (104), B220 (RA3‐6B2), CD45.1 (A20), TCRβ (H57‐597), CD8β (53‐5.8 or YST156.7.7 or eBioH35‐17.2), CD4 (RM4‐5 or GK1.5 or eBioGK1.5), Ter119 (TER‐119), IFN‐γ (XMG 1.2), Siglec H (551), CD11c (N418), IL17A (TC11‐18‐10.1), FoxP3 (FJK‐165), CD103 (M290), Siglec F (E50‐2440), CD64, (X54‐5/7.1), CD135 (A2F10), CD117 (2B8), CD45.2 (104) and XCR1 (ZET) all from eBioscience, BioLegend or BD Biosciences. Antibody to GP2 (2F11 C3) was from MBL.

Flow cytometry was performed according to standard procedures.^37^ Dead cells identified by addition of propidium iodide (Invitrogen), Viability Dye eFluor®450 (eBioScience), or Red or Aqua LIVE/DEAD Fixable Dead Cell Staining Kit (Life Technologies), and cell aggregates (identified on FSC‐A vs FSC‐W scatterplots) were excluded from analyses. Intracellular staining was performed using the FoxP3 Fixation/Permeabilization Kit (eBioscience) according to manufacturer's instructions. Data were acquired on a FACSAriaII or LSRII (BD Biosciences) and analysed using FlowJo software (Tree Star). Sorting was performed on a FACSAriaII or on a MoFlow®Astrios (Beckman Coulter).

### 2.5 Bone marrow chimeras

Mixed bone marrow (BM) chimeras were generated by i.v. injection of CD45.2^+^ *Itgax‐Cre*.*Tgfbr1* ^*fl/fl*^.*Rag1* ^−/−^ or *Tgfbr1* ^*fl/fl*^.*Rag1* ^−/−^ BM^17^ with CD45.1^+^ or CD45.1^+^CD45.2^+^ C57Bl/6 BM at a 1:1 ratio (2 × 10^6^ cells/mouse) into irradiated (900 rad) congenic CD45.1^+^CD45.2^+^ or CD45.1^+^ C57Bl/6 recipients, respectively. Recipients received ciprofloxacin (100 mg/mL, Bayer HealthCare AG) in their drinking water for 3 weeks, and analysis was performed 8 weeks after BM reconstitution.

### 2.6 In vivo treatment with TLR ligands

C57BL/6 mice received R848 (20 μg, InvivoGen) by oral gavage or were injected i.p. with FliC (20 μg, kind gift from Dr. Flores‐Langarica, University of Birmingham, U.K.) or polyI:C (100 μg, Sigma‐Aldrich). MLN were isolated 17 hours after treatment and analysed by flow cytometry.

### 2.7 In vitro cell culture and cytokine measurements

SI‐LP, LI‐LP and MLN cell suspensions were re‐stimulated in vitro with PMA (50 ng/mL, Sigma‐Aldrich) and Ionomycin (500 ng/mL, Sigma‐Aldrich) for 4 hours. At 1 hour of re‐stimulation, cells were treated with Brefeldin A (3 μg/mL, BioLegend). For cDC cultures, FACS‐sorted cDC (2 × 10^4^ cells/well) were incubated in R10 medium, in the presence or absence of LPS (1 μg/mL, Sigma‐Aldrich) or FliC (100 ng/mL;) for 22 hours at 37°C and 5% CO_2_. Cells were analysed by flow cytometry, and levels of IL‐6 in cell supernatants assessed using the cytometric bead array (CBA) flex set kit (BD Bioscience) according to manufacturer's instructions.

### 2.8 Statistical analysis

Statistical significance was determined with Student's *t* test except where indicated and GraphPad Prism software (GraphPad).

## 3 RESULTS

### 3.1 GP2 is selectively expressed by SI‐LP CD103 ^+^ CD11b ^+^ cDC

**FIGURE 1:**
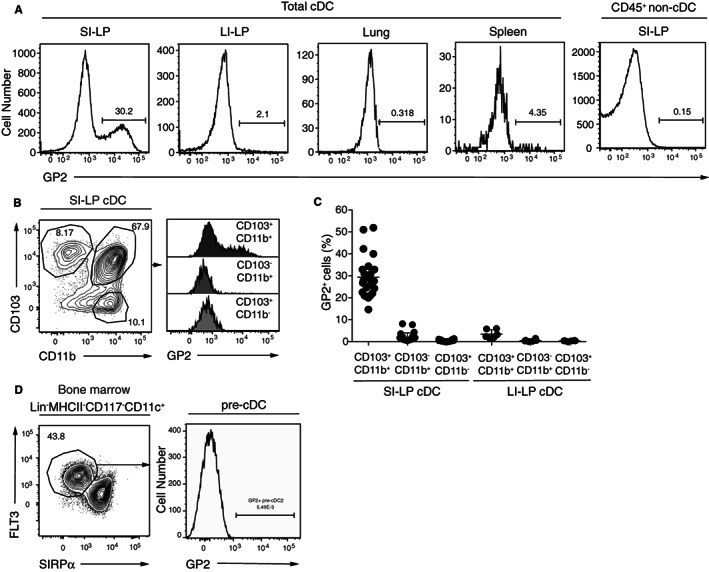
Identification of GP2‐expressing cDC in the SI‐LP. A, Representative flow cytometry analysis of GP2 expression on cDC from indicated organs. SI‐LP, small intestinal lamina propria, LI‐LP, large intestinal lamina propria. B, Representative flow cytometry plot and (C) pooled data of GP2 expression within indicated cDC populations. D, Representative flow cytometry plot of GP2 expression on BM pre‐cDC. Data are from (A) 1 (lung data)—7, (B) 2, (C, D) 2‐7 independent experiments. C, Each symbol represents an individual mouse and line represents mean ± SEM. See also Figure S1

To assess GP2 expression by intestinal cDC, we initially performed flow cytometry on SI‐LP tissue that had been digested with Liberase™ but failed to identify GP2 expressing cDC. To address the possibility that this protocol resulted in the loss of GP2 from the cDC surface, GP2 expression was assessed on cDC that had migrated out from tissue pieces after 20 hours overnight culture in vitro (see Section 2). Under these conditions, GP2 was expressed by a large proportion of SI‐LP cDC, but not other SI‐LP CD45^+^ cells, and by few cDC derived from the large intestinal (LI) LP, lung or spleen (Figure 1A, for gating strategy see Figure S1A,B). To assess whether GP2 was expressed on a particular SI‐LP cDC subset, SI‐LP derived cDC were co‐stained for CD103 and CD11b to identify intestinal cDC1 (CD103^+^CD11b^−^ cDC) and cDC2 (CD103^+^CD11b^+^ and CD103^−^CD11b^+^) (Figure 1B). GP2 was expressed almost exclusively by CD103^+^CD11b^+^ SI cDC2 (Figure 1B,C); however, the proportion of CD103^+^CD11b^+^ cDC expressing GP2 was highly variable between mice (Figure 1C). GP2 was also selectively expressed on LI‐LP derived CD103^+^CD11b^+^ cDC, although at a much lower frequency than observed on SI‐LP derived CD103^+^CD11b^+^ cDC (Figure 1C). In the spleen, GP2 expression was expressed on a minor population of XCR1^+^ cDC1 (Figure S1C,D), suggesting that expression of GP2 is not restricted to the cDC2 lineage. Collagenase II digestion of SI‐LP preserved GP2 expression on CD103^+^CD11b^+^ cDC and resulted in the detection of similar proportions of GP2 expressing CD103^+^CD11b^+^ cDC to that observed using the ‘walk‐out’ isolation procedure (Figure S1E). In contrast, GP2 was barely detected on other SI‐LP CD45^+^ cells after collagenase II digestion, including CD64^+^ macrophages (Figure S1F, data not shown). Importantly, GP2 was not expressed by pre‐cDC (Figure 1E, for pregating see Figure S1G), indicating that GP2 is induced on CD103^+^CD11b^+^ cDC within the SI‐LP environment.

### 3.2 SI‐LP GP2 ^+^ CD103 ^+^ CD11b ^+^ cDC migrate to intestinal‐draining MLN

**FIGURE 2:**
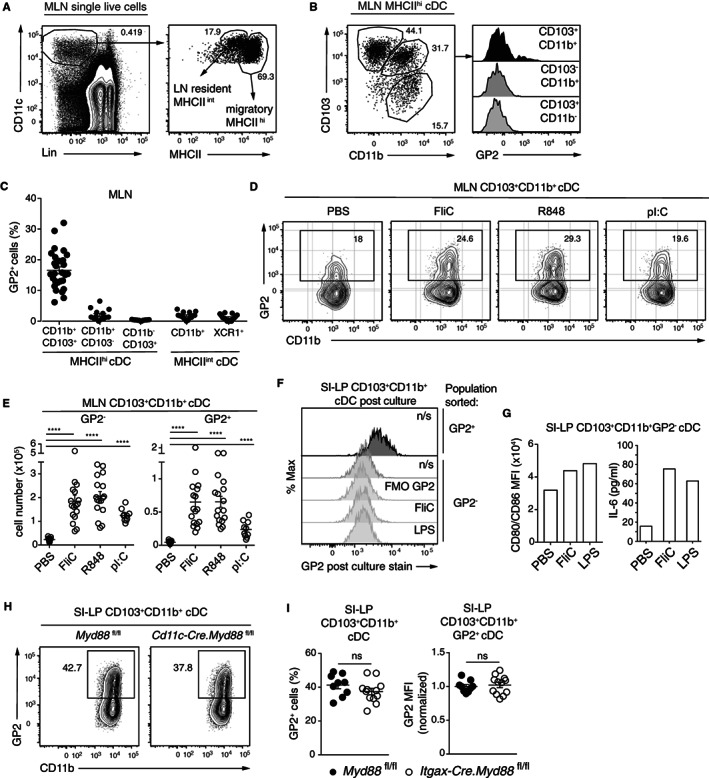
GP2^+^ cDC in MLN in steady state and after in vivo TLR stimulation. A, Representative flow cytometry plots showing the gating strategy to identify migratory and MLN‐resident cDC subsets. Lineage (Lin); B220, CD19, NK1.1 and TCRβ. B, Representative flow cytometry plot gating on migratory MLN cDC (left), and histograms of GP2 expression within indicated cDC population (right). C, Frequency of GP2 expressing cells within the indicated MLN cDC subsets. Each symbol represents an individual mouse. D, Representative flow cytometry plot of GP2 expression, and (E) total number of indicated cDC population in the MLN 17 hours after injection with FliC (20 μg), R848 (20 μg), pI:C (100 μg) or PBS. Each symbol represents an individual mouse. F, Representative histogram of GP2 staining and (G) CD80 and CD86 expression intensities and IL‐6 secretion by sorted CD103^+^CD11b^+^GP2^−^ cDC, 22 hours after in vitro culture with FliC (100 ng/mL), LPS (1 μg/mL), or PBS (n/s, not stimulated). H, Representative GP2 staining and (I) percentage of SI‐LP CD103^+^CD11b^+^ cDC expressing GP2 (left) and median fluorescence intensity (MFI) of GP2 staining on GP2^+^CD103^+^CD11b^+^ SI‐LP cDC in *Myd88* ^*fl/fl*^ and *Itgax‐Cre*.*Myd88* ^*fl/fl*^ mice. I, Each symbol represents an individual mouse. Data are from (A‐C) 7, (D‐E) 4, (F‐G) 1 and (H‐I) 3 independent experiments. (C, E and I) Line represents mean ± SEM. ns, non‐significant, ****P < .0001

SI‐LP cDC migrate to the draining MLN^10^, ^38^, ^39^, ^40^ and can be distinguished in the MLN from MLN‐resident cDC based on higher expression of MHCII^39^ (Figure 2A). In the MLN, GP2 expression was restricted to MHCII^hi^ intestinal‐derived CD103^+^CD11b^+^ cDC2 and was not expressed by MLN‐resident cDC1 or cDC2 (Figure 2B,C). Oral or i.p. administration of TLR ligands induces a rapid migration of SI‐LP‐derived cDC into the MLN.^39^, ^41^ To assess whether TLR ligands could drive the migration of GP2 expressing CD103^+^CD11b^+^ cDC2 from the SI‐LP into the MLN, WT mice were injected i.p. with FliC (TLR5 ligand) or pI:C (TLR3 ligand), or orally with R848 (TLR7 ligand), and accumulation of GP2^+^CD103^+^CD11b^+^ cDC in the MLN assessed 17 hours later. All three TLR ligands elicited an accumulation of both GP2^+^CD103^+^CD11b^+^ and GP2^−^CD103^+^CD11b^+^, cDC2 into the MLN (Figure 2D,E). FliC and LPS failed to induce GP2 expression on flow cytometry cell sorted GP2^−^CD103^+^CD11b^+^ SI‐LP cDC2 in vitro despite inducing an upregulation of CD80/CD86 upregulation and IL‐6 secretion in these cells (Figure 2F,G), suggesting that accumulation of GP2^+^CD103^+^CD11b^+^ cDC to the MLN likely reflects an enhanced mobilization of these cells from the SI‐LP rather than a de novo induction of GP2 on CD103^+^CD11b^+^ cDC2. Consistent with the former, the proportion of GP2‐expressing SI‐LP CD103^+^CD11b^+^ cDC2 and levels of GP2 expression on GP2^+^ cDC2 was similar in *Itgax‐Cre*.*Myd88* ^*fl/fl*^ mice and *Myd88* ^*fl/fl*^ littermates (Figure 2H,I). Thus, GP2 expression by SI‐LP CD103^+^CD11b^+^ cDC2 is independent of MyD88 signalling.

**FIGURE 3:**
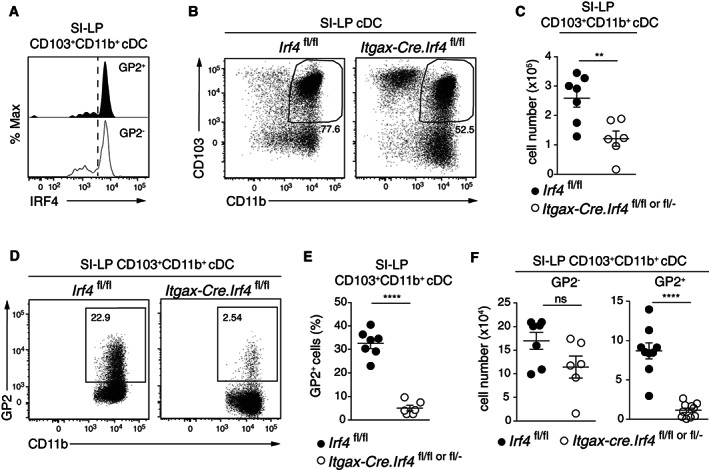
GP2^+^CD103^+^CD11b^+^ cDC are dependent on the transcription factor IRF4. A, Intracellular IRF4 staining within GP2^+^ and GP2^−^ CD103^+^CD11b^+^ SI‐LP cDC. B, Representative flow cytometry plots and (C) total number of CD103^+^CD11b^+^ cDC from the SI‐LP of *Irf4* ^*fl/fl*^ and *Itgax‐Cre*.*Irf4* ^*fl/fl* or *fl/−*^ mice isolated from the ‘walk‐out’ method. D, Representative flow cytometry plots of GP2 expression, (E) percentage of GP2^+^ cells within the CD103^+^CD11b^+^ cDC population and (F) total number of GP2^+^CD103^+^CD11b^+^ and GP2^−^CD103^+^CD11b^+^ cDC in the SI‐LP of *Irf4* ^*fl/fl*^ and *Itgax‐Cre*.*Irf4* ^*fl/fl* or *fl/−*^ mice. A‐F, Data are from 2 to 3 independent experiments, with (C, E and F) each symbol representing and individual mouse and line representing mean ± SEM. **P < .01, ****P < .0001, ns, not significant

### 3.3 GP2 expression on intestinal CD103 ^+^ CD11b ^+^ cDC requires IRF4

Mice whose cDC are deficient in *Irf4* (*Itgax‐Cre*.*Irf4* ^*fl/fl*^ mice) have an almost complete loss of intestinal‐derived CD103^+^CD11b^+^ cDC in MLN and an approximate 50% reduction in SI‐LP CD103^+^CD11b^+^ cDC.^5^, ^7^ IRF4 was expressed at similar levels by GP2^+^ and GP2^−^ SI‐LP CD103^+^CD11b^+^ cDC (Figure 3A), and consistent with previous findings,^5^ SI‐LP CD103^+^CD11b^+^ cDC numbers were reduced in *Itgax‐Cre*.*Irf4* ^*fl/fl* or *fl/−*^ mice (Figure 3B,C). Interestingly, the proportion of GP2 expressing CD103^+^CD11b^+^ cDC in the SI‐LP of *Itgax‐Cre*.*Irf4* ^*fl/fl or fl/−*^ mice was reduced compared with *Irf4* ^*fl/fl*^ littermates (Figure 3D,E), resulting in an almost complete loss of GP2 expressing cDC2 in these mice (Figure 3F). Thus, IRF4 is required for the generation and/or maintenance of GP2 expressing SI‐LP CD103^+^CD11b^+^ cDC2.

### 3.4 Retinoic acid signalling in intestinal cDC2 is required for optimal expression of GP2

**FIGURE 4:**
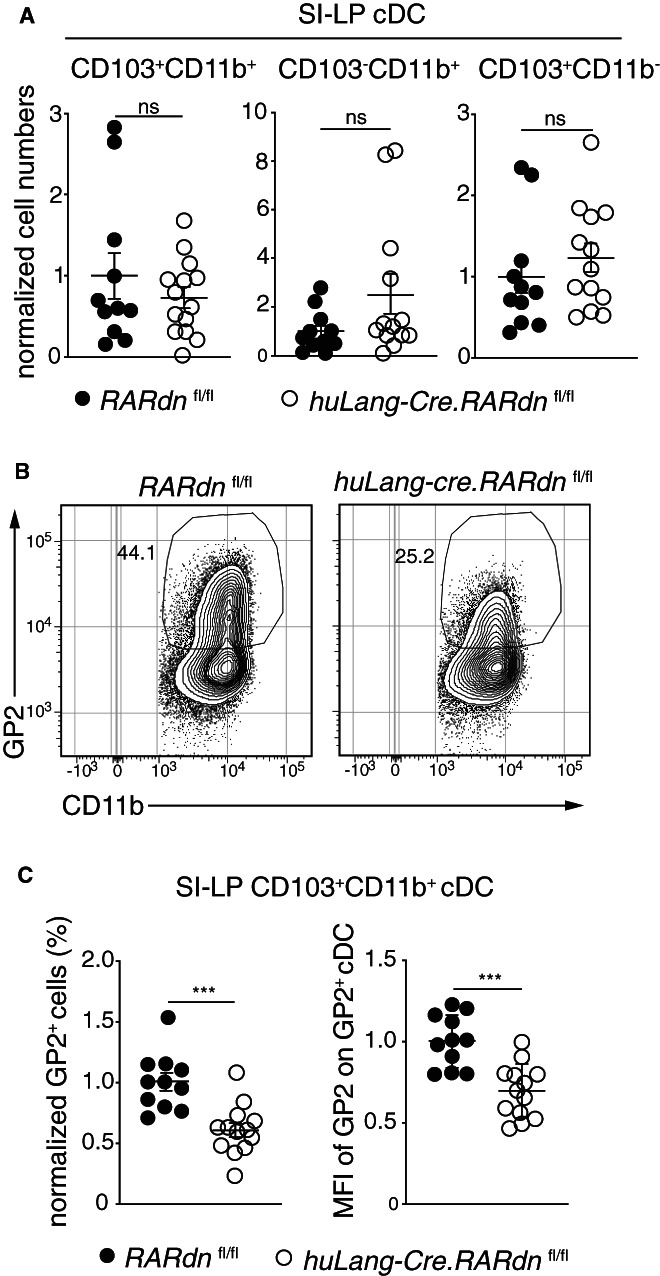
Intrinsic retinoic acid signalling promotes GP2 expression in CD103^+^CD11b^+^ cDC. A, Number of indicated cDC subsets in SI‐LP of *RARdn* ^*fl/fl*^ and *hu‐Lang‐Cre*.*RARdn* ^*fl/fl*^ mice normalized to the mean of cell numbers from the *RARdn* ^*fl/fl*^ group. B, Representative GP2 staining and (C) percentage of SI‐LP CD103^+^CD11b^+^ cDC expressing GP2 (left panel) and median fluorescence intensity (MFI) of GP2 staining on GP2^+^CD103^+^CD11b^+^ SI‐LP cDC cDC (right panel) in *RARdn* ^*fl/fl*^ and *hu‐Lang‐cre*.*RARdn* ^*fl/fl*^ mice normalized to the mean MFI of the the *RARdn* ^*fl/fl*^ group. A‐C, Data are from 4 experiments with each symbol representing an individual mouse and line representing mean ± SEM. ***P < .001, ns, not significant. See also Figure S2

The vitamin A metabolite, retinoic acid (RA), is an important regulator of intestinal homeostasis and has been implicated in intestinal cDC differentiation.^42^, ^43^ To determine whether RA signalling in SI‐LP CD103^+^CD11b^+^ cDC modulates their expression of GP2, we generated *huLang‐Cre*.*RARdn* mice whose SI‐LP CD103^+^CD11b^+^ cDC constitutively express a dominant negative form of the retinoic acid receptor.^34^, ^44^, ^45^ The number of SI‐LP CD103^+^CD11b^+^, CD103^−^CD11b^+^ and CD103^+^CD11b^−^ cDC and their migratory counterparts in the MLN were similar in *huLang‐Cre*.*RARdn* and *RARdn* littermates (Figure 4A and Figure S2A), indicating that RA signalling is not required for the maintenance or survival of intestinal CD103^+^CD11b^+^ cDC. However, the proportion of SI‐LP and MLN CD103^+^CD11b^+^ cDC that expressed GP2 and the levels of GP2 on SI‐LP GP2^+^ cDC was reduced *Lang‐Cre*.*RARdn* mice (Figure 4B,C and Figure S2B). Thus, RA signalling in SI‐LP CD103^+^CD11b^+^ cDC is required for their optimal expression of GP2.

### 3.5 TGFβ directly suppresses GP2 expression on intestinal cDC2

**FIGURE 5:**
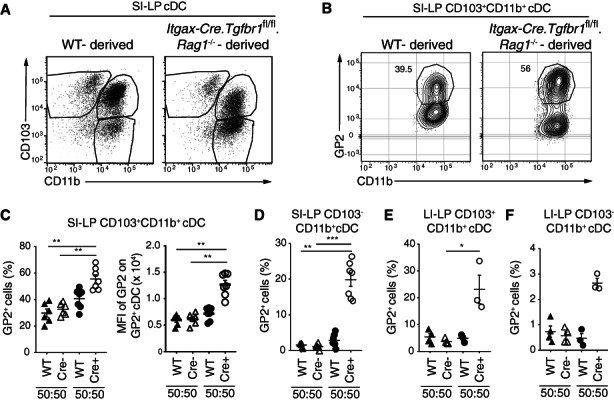
TGFβR1‐signalling suppresses GP2 expression on intestinal cDC2. A, Representative flow cytometry plots of SI‐LP cDC deriving from WT or *Itgax‐Cre*.*Tgfbr1* ^*fl/fl*^.*Rag1* ^−/−^ BM from the same mixed BM chimeric mouse. B, Representative GP2 expression, (C‐F) frequency of GP2^+^ cells amongst SI‐LP (C) CD103^+^CD11b^+^ cDC (left panel) (D) CD103^−^CD11b^+^, and LI‐LP (E) CD103^+^CD11b^+^ and (F) CD103^−^CD11b^+^ cDC, and (C) MFI of GP2 expression on GP2^+^CD103^+^CD11b^+^ SI‐LP cDC derived from indicated BM in mixed BM chimeras. C‐F, Each symbol represents an independent chimera and line represents mean ± SEM. *P < .05, **P < .01, ***P < .001, as assessed using Kruskal‐Wallis. See also Figure S3

TGFβ signalling in cDC has been suggested to play an important role in the generation of SI‐LP CD103^+^CD11b^+^ cDCs.^17^ To determine the role of TGFβR1‐signalling in the generation of GP2^+^ SI‐LP cDC2, BM from CD45.1^+^ WT and either CD45.2^+^ *Itgax‐Cre*.*Tgfbr1* ^*fl/fl*^.*Rag1* ^−/−^ (Cre^+^) or control CD45.2^+^ *Tgfβr1* ^*fl/fl*^.*Rag1* ^*−/−*^ (Cre^−^) was injected at a 1:1 ratio into irradiated CD45.1^+^CD45.2^+^ recipients and the SI‐LP cDC compartment of recipient mice assessed 8 weeks post‐transfer. Consistent with previous findings,^17^ SI‐LP CD103^+^CD11b^+^ cDC deriving from *Itgax‐Cre*.*Tgfbr1* ^*fl/fl*^.*Rag1* ^−/−^ BM had reduced levels of CD103 (Figure 5A). In contrast, a larger proportion of SI‐LP CD103^+^CD11b^+^ cDC derived from *Itgax‐Cre*.*Tgfbr1* ^*fl/fl*^.*Rag1* ^−/−^ BM expressed GP2 and at higher levels compared with CD103^+^CD11b^+^ cDC derived from WT or *Tgfbr1* ^*fl/fl*^.*Rag1* ^−/−^ BM (Figure 5B,C). GP2 was also expressed by a significant proportion of SI‐LP CD103^−^CD11b^+^ cDC deriving from *Itgax‐Cre*.*Tgfbr1* ^*fl/fl*^.*Rag1* ^−/−^ but not WT or *Tgfbr1* ^*fl/fl*^.*Rag1* ^−/−^ BM (Figure 5D). Furthermore, in contrast to LI‐LP CD103^+^CD11b^+^ cDC that derived from WT or *Tgfbr1* ^*fl/fl*^.*Rag1* ^−/−^ BM, a large proportion of LI‐LP CD103^+^CD11b^+^ cDC that derived from *Itgax‐Cre*.*Tgfbr1* ^*fl/fl*^.*Rag1* ^−/−^ BM expressed GP2 (Figure 5E), as did a small proportion of LI‐LP CD103^−^CD11b^+^ cDC2 (Figure 5F). GP2 was not expressed on SI‐LP or LI‐LP CD103^+^CD11b^−^ cDC1 that derived from *Itgax‐Cre*.*Tgfbr1* ^*fl/fl*^.*Rag1* ^−/−^ BM (Figure S3A). Collectively, these results suggest that TGFβ signalling in intestinal cDC2 suppresses their expression of GP2.

### 3.6 GP2 expression on intestinal CD103 ^+^ CD11b ^+^ cDC2 is not required for intestinal immune homeostasis

**FIGURE 6:**
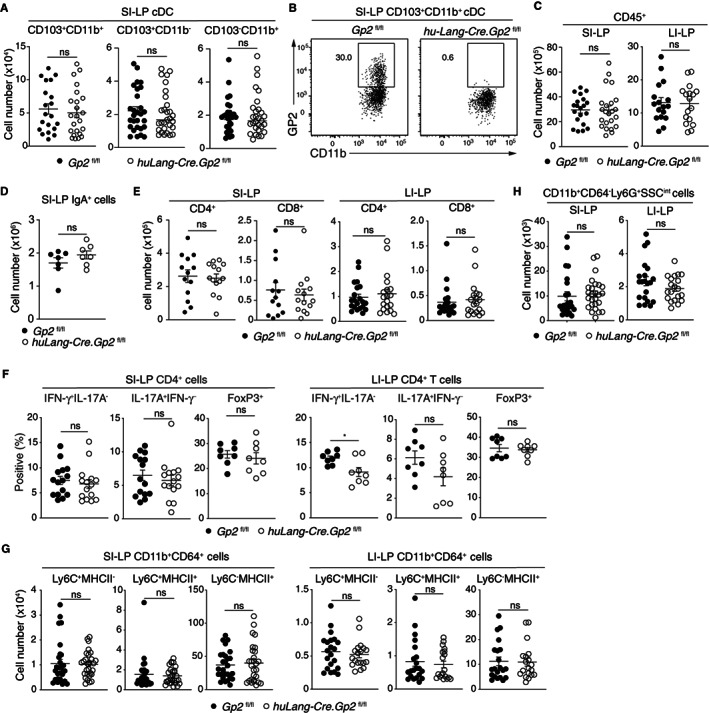
Impact of GP2 deletion in CD103^+^CD11b^+^ cDC on intestinal immune compartments in the LP. A, Total number of SI‐LP cDC subsets and (B) representative GP2‐staining of SI‐LP CD103^+^CD11b^+^ cDC in *Gp2* ^*fl/fl*^ and *hu‐Lang‐Cre*.*Gp2* ^*fl/fl*^ mice. C, Total number of CD45^+^ cells, (D) IgA^+^ Plasma cells, (E) CD4^+^ and CD8^+^ T cells, (F) proportion of IFN‐γ^+^IL17A^−^, IL17A^+^IFN‐γ^−^ and FoxP3^+^ Tregs amongst CD4^+^ T cells, (G) number of monocyte‐derived subsets and (H) neutrophils in the SI‐LP and LI‐LP in *Gp2* ^*fl/fl*^ and *hu‐Lang‐Cre*.*Gp2* ^*fl/fl*^ mice. Data are pooled from (A, D‐H) 2‐5 and independent experiments. Each symbol represents an independent mouse and line represents mean ± SEM. *P < .05, ns, not significant. See also Figure S4

GP2 has been suggested to modulate innate and adaptive immune cell responses.^26^, ^46^, ^47^, ^48^, ^49^, ^50^ To determine whether GP2 expression by intestinal CD103^+^CD11b^+^ cDC2 was important for intestinal immune homeostasis, *huLang‐Cre* mice were crossed with *Gp2* ^*fl/fl*^ to generate *huLang‐Cre*.*Gp2* ^*fl/fl*^ and *Gp2* ^*fl/fl*^ littermates. *huLang‐Cre*.*Gp2* ^*fl/fl*^ mice had similar numbers of CD103^−^CD11b^+^ and CD103^+^CD11b^+^ cDC2 and CD103^+^CD11b^−^ cDC1 in the SI‐LP, LI‐LP and MLN as *Gp2* ^*fl/fl*^ littermate controls (Figure 6A and Figure S4A,B). As expected, SI‐LP and MLN CD103^+^CD11b^+^ cDC in *huLang‐Cre*.*Gp2* ^*fl/fl*^ mice lacked expression of GP2 (Figure 6B and Figure S4C).

To determine whether absence of GP2 on intestinal CD103^+^CD11b^+^ cDC2 impacted on intestinal immune cell homeostasis, we assessed the intestinal adaptive and innate immune composition of *huLang‐Cre*.*Gp2* ^*fl/fl*^ and *Gp2* ^*fl/fl*^ littermates. *HuLang‐Cre*.*Gp2* ^*fl/fl*^ and *Gp2* ^*fl/fl*^ littermates had similar numbers of CD45^+^ cells in both SI‐LP and LI‐LP (Figure 6C). Similarly, the total number of IgA^+^ plasma cells (Figure 6D), CD4^+^ and CD8^+^ T cells (Figure 6E) as well as the proportions of IFN‐γ^+^, IL‐17^+^ and FoxP3^+^ cells amongst SI‐LP and LI‐LP CD4^+^ T cells did not differ in the between *huLang‐Cre*.*Gp2* ^*fl/fl*^ and *Gp2* ^*fl/fl*^ littermates (Figure 6F, for gating strategy see Figure S4D). Regarding innate immune cell subsets, the SI‐LP and LI‐LP of *huLang‐Cre*.*Gp2* ^*fl/fl*^ mice contained similar numbers of tissue‐resident macrophages (CD64^+^CD11b^+^Ly6C^−^MHCII^+^), monocytes (CD64^+^CD11b^+^Ly6C^++^MHCII^−^), monocyte intermediates (CD64^+^CD11b^+^Ly6C^+^MHCII^+^) and neutrophils as *Gp2* ^*fl/fl*^ littermates (Figure 6G,H), the latter indicating that absence of GP2 on cDC2 does not result in low‐grade intestinal inflammation. Collectively, these results suggest that GP2 expression by CD103^+^CD11b^+^ cDC is not required for intestinal immune homeostasis.

## 4 DISCUSSION

In the current study, we demonstrate that a large proportion of SI‐LP CD103^+^CD11b^+^ cDC2 express the zymogen granule membrane protein GP2. GP2 was not expressed on pre‐DC, cDC2 in the lung, spleen or LI‐LP suggesting local induction in the SI‐LP. Consistent with this, we demonstrate a local and direct role for the vitamin A metabolite, RA, in inducing GP2 expression on small intestinal CD103^+^CD11b^+^ cDC2 in vivo. In contrast, cell‐intrinsic absence of TGFβ signalling resulted in enhanced GP2 expression on CD103^+^CD11b^+^ cDC2 and upregulation of GP2 on additional SI‐LP and LI‐LP cDC2 populations. SI‐LP‐derived GP2^+^CD103^+^CD11b^+^ cDC2 were present in the MLN, and while GP2 expression on these cells was independent of Myd88 signalling, migration of GP2^+^CD103^+^CD11b^+^ cDC2 to the MLN was dramatically enhanced following administration of TLR agonists. Finally, mice whose CD103^+^CD11b^+^ cDC2 were deficient in GP2 had normal immune cell composition and numbers in LI‐LP, SI‐LP and MLN suggesting that cDC2‐derived GP2 is not required for maintaining intestinal homeostasis.

We have previously shown that *Irf4* is required for the survival of SI‐LP CD103^+^CD11b^+^ cDC2; its absence leading to an approximate 50% reduction in these cells.^5^, ^7^ Interestingly, we here show that GP2 expressing SI‐LP CD103^+^CD11b^+^ cDC population but not GP2^−^CD103^+^CD11b^+^ cDC are reduced in the absence of *Irf4*. A likely explanation for these results is that IRF4, in addition to promoting CD103^+^CD11b^+^ cDC2 survival, is required for the induction of GP2 on these cells, increasing the numbers of GP2^−^CD103^+^CD11b^+^ cDC2 in *Itgax‐Cre*.*Irf4* ^*fl/fl*^ mice. Alternatively, GP2^+^CD103^+^CD11b^+^ cDC2 as a population may be more dependent on IRF4 for their survival compared with their GP2‐negative counterparts. Future studies comparing the transcriptome of GP2^−^ and GP2^+^CD103^+^CD11b^+^ cDC may help resolve these possibilities.

The absence of GP2 on cDC precursors, on extra‐intestinal cDC2 and expression of GP2 on an only minor fraction of CD103^+^CD11b^+^ LI‐LP cDC2 strongly indicates that GP2 is induced on CD103^+^CD11b^+^ cDC2 by factors within the SI‐LP environment. Here, we identify a role for retinoic acid signalling in cDC in inducing GP2 expression in SI‐LP CD103^+^CD11b^+^ cDC2. Of note, we have previously shown retinol levels to be higher in the SI compared with the LI and that SI‐LP cDC receive increased RA signals in the steady state compared with their counterparts in the LI‐LP.^13^ Thus, enhanced RA signalling in the SI may in part explain the increased expression of GP2 on SI‐LP compared with LI‐LP CD103^+^CD11b^+^ cDC2. Why GP2 is not expressed by closely related CD103^−^CD11b^+^ cDC2, or by CD103^+^CD11b^−^ cDC1 in the SI‐LP remains unclear. One potential explanation is that these populations receive reduced RA signalling in situ compared with CD103^+^CD11b^+^ cDC2; however, we think this unlikely since SI‐LP CD103+CD11b‐ cDC1 are themselves a major source of RA.^15^ Alternatively, as discussed below, these populations may in part receive local signals that inhibit expression of GP2.

Cell intrinsic TGFβR signalling has been suggested to play a key role in the development of SI‐LP CD103^+^CD11b^+^ cDC2 from CD103^−^CD11b^+^ cDC2 intermediates.^17^ We therefore hypothesized that TGFβR may also be required for the induction of GP2 on CD103^+^CD11b^+^ cDC2. Surprisingly, while SI‐LP CD103^+^CD11b^+^ cDC2 showed an expected reduction in CD103 expression, the proportion of GP2 expressing CD103^+^CD11b^+^ cDC2 as well as the levels of GP2 expression on these cells increased in the absence of TGFβR signalling. Moreover, while in the absence of TGFβR signalling SI‐LP and LI‐LP CD103^+^CD11b^−^ cDC remained GP2^−^, a proportion of SI‐LP CD103^−^CD11b^+^ cDC2 expressed GP2, as did colon CD103^+^CD11b^+^ and CD103^−^CD11b^+^ cDC2, albeit at lower levels compared with their corresponding populations in the SI‐LP. Thus, TGFβR signalling acts to curtail GP2 expression on cDC2 in both the SI‐LP and LI‐LP. Why intestinal CD103^−^CD11b^+^ cDC2, whose expression of GP2 appears to be suppressed in vivo by TGFβ, do not upregulate CD103 in response to such signals remains unclear. Interestingly, despite our finding the GP2 is expressed by a subset of splenic cDC1, and thus not restricted to a specific cDC subset, absence of TGFβR signalling failed to induce GP2 expression on SI‐LP and LI‐LP cDC1. Collectively, our findings suggest that GP2 expression by cDC is regulated by a complex interplay of local environmental factors.

Our observation that GP2 is expressed on a subset of SI‐LP‐derived CD103^+^CD11b^+^ cDC in the MLN, and these cells increase in number following in vivo TLR stimulation indicates that cDC‐derived GP‐2 may play a role in the regulation of intestinal adaptive immune responses. Consistent with this possibility, GP2 has been implicated in modulating T cell responses.^26^ Despite this, we did not observe alterations in intestinal adaptive immune cell numbers or proportions in mice whose intestinal cDC lacked GP2, demonstrating that GP2 expression by intestinal cDC is not required for the establishment and maintenance of this compartment in the steady state. Importantly, however, our studies were performed on mice housed in pathogen‐free conditions and in non‐challenged situations, and it will be interesting in future studies to explore the role of cDC‐derived GP2 in models of intestinal infection and inflammation. In this regard, GP2 contains protease cleavage sites,^51^ and we speculate that inflammation‐driven induction of metalloproteases^52^, ^53^ might result in the release of GP2 from the cDC surface into the LP where it could possibly act as a soluble immunomodulatory mediator.

In summary, we demonstrate that GP2 is expressed by a large population of SI‐LP CD103^+^CD11b^+^ cDC and that expression of GP2 by intestinal cDC is regulated by local environmental signals including RA and TGFβ. Further studies are required to address whether GP2^+^ and GP2^−^ CD103^+^CD11b^+^ cDC represent developmentally and functionally distinct cDC subsets, or different activation stages of the same subset as well as the importance of CD103^+^CD11b^+^ cDC‐derived GP2 in intestinal infection and inflammation.

## AUTHORS CONTRIBUTION

WWA and KML conceived and designed the study. KML, CDS, FA and KK performed experiments and analysed the data. HO and AM provided key reagents. KML and WWA wrote the manuscript with input from all authors.

## CONFLICT OF INTEREST

The authors declare no commercial or financial conflict of interest.

## Supporting information


Figure S1.

Figure S2.

Figure S3.

Figure S4.

## Data Availability

The data that support the findings of this study are available from the corresponding author upon reasonable request.

## References

[1] Cerovic V , Houston SA , Westlund J , et al. Lymph‐borne CD8alpha+ dendritic cells are uniquely able to cross‐prime CD8+ T cells with antigen acquired from intestinal epithelial cells. Mucosal Immunol. 2015;8:38‐48. 24850430 10.1038/mi.2014.40 PMC4156465

[2] Joeris T , Gomez‐Casado C , Holmkvist P , et al. Intestinal cDC1 drive cross‐tolerance to epithelial‐derived antigen via induction of FoxP3(+)CD8(+) Tregs. Sci Immunol. 2021;6:eabd3774. 34088744 10.1126/sciimmunol.abd3774

[3] Luda KM , Joeris T , Persson EK , et al. IRF8 transcription‐factor‐dependent classical dendritic cells are essential for intestinal T cell homeostasis. Immunity. 2016;44:860‐874. 27067057 10.1016/j.immuni.2016.02.008

[4] Ohta T , Sugiyama M , Hemmi H , et al. Crucial roles of XCR1‐expressing dendritic cells and the XCR1‐XCL1 chemokine axis in intestinal immune homeostasis. Sci Rep. 2016;6:23505. 27005831 10.1038/srep23505 PMC4804307

[5] Persson EK , Uronen‐Hansson H , Semmrich M , et al. IRF4 transcription‐factor‐dependent CD103(+)CD11b(+) dendritic cells drive mucosal T helper 17 cell differentiation. Immunity. 2013;38:958‐969. 23664832 10.1016/j.immuni.2013.03.009

[6] Satpathy AT , Briseno CG , Lee JS , et al. Notch2‐dependent classical dendritic cells orchestrate intestinal immunity to attaching‐and‐effacing bacterial pathogens. Nat Immunol. 2013;14:937‐948. 23913046 10.1038/ni.2679 PMC3788683

[7] Schlitzer A , McGovern N , Teo P , et al. IRF4 transcription factor‐ dependent CD11b(+) dendritic cells in human and mouse control mucosal IL‐17 cytokine responses. Immunity. 2013;38:970‐983. 23706669 10.1016/j.immuni.2013.04.011 PMC3666057

[8] Demiri M , Muller‐Luda K , Agace WW , Svensson‐Frej M . Distinct DC subsets regulate adaptive Th1 and 2 responses during *Trichuris muris* infection. Parasite Immunol. 2017;39. doi:10.1111/pim.12458 28802050

[9] Denning TL , Norris BA , Medina‐Contreras O , et al. Functional specializations of intestinal dendritic cell and macrophage subsets that control Th17 and regulatory T cell responses are dependent on the T cell/APC ratio, source of mouse strain, and regional localization. J Immunol. 2011;187:733‐747. 21666057 10.4049/jimmunol.1002701 PMC3131424

[10] Houston SA , Cerovic V , Thomson C , Brewer J , Mowat AM , Milling S . The lymph nodes draining the small intestine and colon are anatomically separate and immunologically distinct. Mucosal Immunol. 2016;9:468‐478. 26329428 10.1038/mi.2015.77

[11] Mayer JU , Demiri M , Agace WW , MacDonald AS , Svensson‐Frej M , Milling SW . Different populations of CD11b(+) dendritic cells drive Th2 responses in the small intestine and colon. Nat Commun. 2017;8:15820. 28598427 10.1038/ncomms15820 PMC5472728

[12] Agace WW , Persson EK . How vitamin a metabolizing dendritic cells are generated in the gut mucosa. Trends Immunol. 2012;33:42‐48. 22079120 10.1016/j.it.2011.10.001

[13] Jaensson‐Gyllenback E , Kotarsky K , Zapata F , et al. Bile retinoids imprint intestinal CD103+ dendritic cells with the ability to generate gut‐tropic T cells. Mucosal Immunol. 2011;4:438‐447. 21289617 10.1038/mi.2010.91 PMC3130189

[14] Johansson‐Lindbom B , Svensson M , Wurbel MA , Malissen B , Marquez G , Agace W . Selective generation of gut tropic T cells in gut‐associated lymphoid tissue (GALT): requirement for GALT dendritic cells and adjuvant. J Exp Med. 2003;198:963‐969. 12963696 10.1084/jem.20031244 PMC2194196

[15] Luda KM , Joeris T , Persson EK , et al. IRF8 dependent classical dendritic cells are essential for intestinal T cell homeostasis. Eur J Immunol. 2016;46:945. 10.1016/j.immuni.2016.02.008 27067057

[16] Svensson M , Marsal J , Ericsson A , et al. CCL25 mediates the localization of recently activated CD8alphabeta(+) lymphocytes to the small‐intestinal mucosa. J Clin Invest. 2002;110:1113‐1121. 12393847 10.1172/JCI15988 PMC150799

[17] Bain CC , Montgomery J , Scott CL , et al. TGF beta R signalling controls CD103(+)CD11b(+) dendritic cell development in the intestine. Nat Commun. 2017;8:620. 28931816 10.1038/s41467-017-00658-6 PMC5607002

[18] Dittie A , Kern HF . The major zymogen granule membrane‐protein Gp‐2 in the rat pancreas is not involved in granule formation. Eur J Cell Biol. 1992;58:243‐258. 1385123

[19] Hoops TC , Rindler MJ . Isolation of the Cdna‐encoding Glycoprotein‐2 (Gp‐2), the major zymogen granule membrane‐protein – homology to uromodulin Tamm‐Horsfall protein. J Biol Chem. 1991;266:4257‐4263. 1999417

[20] Lowe AW , Luthen RE , Wong SME , Grendell JH . The level of the zymogen granule protein Gp2 is elevated in a rat model for acute‐pancreatitis. Gastroenterology. 1994;107:1819‐1827. 7525398 10.1016/0016-5085(94)90826-5

[21] Ronzio RA , Kronquist KE , Lewis DS , Macdonald RJ , Mohrlok SH , Odonnell JJ . Glycoprotein synthesis in adult rat pancreas.4. Subcellular‐distribution of membrane glycoproteins. Biochim Biophys Acta. 1978;508:65‐84. 629968 10.1016/0005-2736(78)90189-x

[22] Hase K , Kawano K , Nochi T , et al. Uptake through glycoprotein 2 of FimH(+) bacteria by M cells initiates mucosal immune response. Nature. 2009;462:226‐230. 19907495 10.1038/nature08529

[23] Terahara K , Yoshida M , Igarashi O , et al. Comprehensive gene expression profiling of Peyer's patch M cells, villous M‐like cells, and intestinal epithelial cells. J Immunol. 2008;180:7840‐7846. 18523247 10.4049/jimmunol.180.12.7840

[24] Yu S , Lowe AW . The pancreatic zymogen granule membrane protein, GP2, binds *Escherichia coli* type 1 fimbriae. BMC Gastroenterol. 2009;9:58. 19627615 10.1186/1471-230X-9-58 PMC2726147

[25] Kurashima Y , Kigoshi T , Murasaki S , et al. Pancreatic glycoprotein 2 is a first line of defense for mucosal protection in intestinal inflammation. Nat Commun. 2021;12:1067. 33594081 10.1038/s41467-021-21277-2 PMC7887276

[26] Werner L , Paclik D , Fritz C , Reinhold D , Roggenbuck D , Sturm A . Identification of pancreatic glycoprotein 2 as an endogenous immunomodulator of innate and adaptive immune responses. J Immunol. 2012;189:2774‐2783. 22891285 10.4049/jimmunol.1103190

[27] Juste C , Kreil DP , Beauvallet C , et al. Bacterial protein signals are associated with Crohn's disease. Gut. 2014;63:1566‐1577. 24436141 10.1136/gutjnl-2012-303786 PMC4173658

[28] Michaels MA , Jendrek ST , Korf T , et al. Pancreatic autoantibodies against CUZD1 and GP2 are associated with distinct clinical phenotypes of Crohn's disease. Inflamm Bowel Dis. 2015;21:2864‐2872. 26273818 10.1097/MIB.0000000000000564

[29] Papp M , Sipeki N , Tornai T , et al. Rediscovery of the anti‐pancreatic antibodies and evaluation of their prognostic value in a prospective clinical cohort of Crohn's patients: the importance of specific target antigens [GP2 and CUZD1]. J Crohns Colitis. 2015;9:659‐668. 25968583 10.1093/ecco-jcc/jjv087

[30] Roggenbuck D , Hausdorf G , Martinez‐Gamboa L , et al. Identification of GP2, the major zymogen granule membrane glycoprotein, as the autoantigen of pancreatic antibodies in Crohn's disease. Gut. 2009;58:1620‐1628. 19549613 10.1136/gut.2008.162495

[31] Somma V , Ababneh H , Ababneh A , et al. The novel Crohn's disease marker anti‐GP2 antibody is associated with ileocolonic location of disease. Gastroenterol Res Pract. 2013;2013:683824. 23762038 10.1155/2013/683824 PMC3671301

[32] Caton ML , Smith‐Raska MR , Reizis B . Notch‐RBP‐J signaling controls the homeostasis of CD8(−) dendritic cells in the spleen. J Exp Med. 2007;204:1653‐1664. 17591855 10.1084/jem.20062648 PMC2118632

[33] Klein U , Casola S , Cattoretti G , et al. Transcription factor IRF4 controls plasma cell differentiation and class‐switch recombination. Nat Immunol. 2006;7:773‐782. 16767092 10.1038/ni1357

[34] Rajaii F , Bitzer ZT , Xu Q , Sockanathan S . Expression of the dominant negative retinoid receptor, RAR403, alters telencephalic progenitor proliferation, survival, and cell fate specification. Dev Biol. 2008;316:371‐382. 18329011 10.1016/j.ydbio.2008.01.041

[35] Kaplan DH , Li MO , Jenison MC , Shlomchik WD , Flavell RA , Shlomchik MJ . Autocrine/paracrine TGF beta 1 is required for the development of epidermal Langerhans cells. J Exp Med. 2007;204:2545‐2552. 17938236 10.1084/jem.20071401 PMC2118472

[36] Hou B , Reizis B , DeFranco AL . Toll‐like receptors activate innate and adaptive immunity by using dendritic cell‐intrinsic and ‐extrinsic mechanisms. Immunity. 2008;29:272‐282. 18656388 10.1016/j.immuni.2008.05.016 PMC2847796

[37] Cossarizza A , Chang HD , Radbruch A , et al. Guidelines for the use of flow cytometry and cell sorting in immunological studies (second edition). Eur J Immunol. 2019;49:1457‐1973. 31633216 10.1002/eji.201970107 PMC7350392

[38] Cerovic V , Houston SA , Scott CL , et al. Intestinal CD103(−) dendritic cells migrate in lymph and prime effector T cells. Mucosal Immunol. 2013;6:104‐113. 22718260 10.1038/mi.2012.53

[39] Hagerbrand K , Westlund J , Yrlid U , Agace W , Johansson‐Lindbom B . MyD88 signaling regulates steady‐state migration of intestinal CD103(+) dendritic cells independently of TNF‐alpha and the gut microbiota. J Immunol. 2015;195:2888‐2899. 26259586 10.4049/jimmunol.1500210

[40] Schulz O , Jaensson E , Persson EK , et al. Intestinal CD103(+), but not CX3CR1(+), antigen sampling cells migrate in lymph and serve classical dendritic cell functions. J Exp Med. 2009;206:3101‐3114. 20008524 10.1084/jem.20091925 PMC2806467

[41] Flores‐Langarica A , Marshall JL , Hitchcock J , et al. Systemic flagellin immunization stimulates mucosal CD103(+) dendritic cells and drives Foxp3(+) regulatory T cell and IgA responses in the mesenteric lymph node. J Immunol. 2012;189:5745‐5754. 23152564 10.4049/jimmunol.1202283

[42] Klebanoff CA , Spencer SP , Torabi‐Parizi P , et al. Retinoic acid controls the homeostasis of pre‐cDC‐derived splenic and intestinal dendritic cells. J Exp Med. 2013;210:1961‐1976. 23999499 10.1084/jem.20122508 PMC3782040

[43] Zeng R , Bscheider M , Lahl K , Lee M , Butcher EC . Generation and transcriptional programming of intestinal dendritic cells: essential role of retinoic acid. Mucosal Immunol. 2016;9:183‐193. 26129652 10.1038/mi.2015.50 PMC4698111

[44] Welty NE , Staley C , Ghilardi N , Sadowsky MJ , Igyarto BZ , Kaplan DH . Intestinal lamina propria dendritic cells maintain T cell homeostasis but do not affect commensalism. J Exp Med. 2013;210:2011‐2024. 24019552 10.1084/jem.20130728 PMC3782055

[45] Pino‐Lagos K , Guo Y , Brown C , et al. A retinoic acid‐dependent checkpoint in the development of CD4+ T cell‐mediated immunity. J Exp Med. 2011;208:1767‐1775. 21859847 10.1084/jem.20102358 PMC3171100

[46] Darisipudi MN , Thomasova D , Mulay SR , et al. Uromodulin triggers IL‐1 beta‐dependent innate immunity via the NLRP3 inflammasome. J Am Soc Nephrol. 2012;23:1783‐1789. 22997256 10.1681/ASN.2012040338 PMC3482735

[47] Liu Y , El‐Achkar TM , Wu XR . Tamm‐Horsfall protein regulates circulating and renal cytokines by affecting glomerular filtration rate and acting as a urinary cytokine trap. J Biol Chem. 2012;287:16365‐16378. 22451664 10.1074/jbc.M112.348243 PMC3351356

[48] Micanovic R , Chitteti BR , Dagher PC , et al. Tamm‐Horsfall protein regulates granulopoiesis and systemic neutrophil homeostasis. J Am Soc Nephrol. 2015;26:2172‐2182. 25556169 10.1681/ASN.2014070664 PMC4552115

[49] Rhodes DCJ , Hinsman EJ , Rhodes JA . Tamm‐Horsfall glycoprotein binds igg with high‐affinity. Kidney Int. 1993;44:1014‐1021. 8264130 10.1038/ki.1993.343

[50] Saemann MD , Weichhart T , Zeyda M , et al. Tamm‐Horsfall glycoprotein links innate immune cell activation with adaptive immunity via a toll‐like receptor‐4dependent mechanism. J Clin Investig. 2005;115:468‐475. 15650774 10.1172/JCI22720 PMC544039

[51] Fritz BA , Lowe AW . Polarized GP2 secretion in MDCK cells via GPI targeting and apical membrane‐restricted proteolysis. Am J Physiol. 1996;270:G176‐G183. 8772516 10.1152/ajpgi.1996.270.1.G176

[52] Lopez‐Boado YS , Wilson CL , Hooper LV , Gordon JI , Hultgren SJ , Parks WC . Bacterial exposure induces and activates matrilysin in mucosal epithelial cells. J Cell Biol. 2000;148:1305‐1315. 10725342 10.1083/jcb.148.6.1305 PMC2174301

[53] Rodrigues DM , Sousa AJ , Hawley SP , et al. Matrix metalloproteinase 9 contributes to gut microbe homeostasis in a model of infectious colitis. BMC Microbiol. 2012;12:105. 22694805 10.1186/1471-2180-12-105 PMC3676156

