# Supplementary figures and images for: Identification and characterization of murine glycoprotein 2‐expressing intestinal dendritic cells
Source: Scand J Immunol. 2022 Oct 17;96(5):e13219. doi: 10.1111/sji.13219 (PMC9786990; doi:10.1111/sji.13219)

Supplementary Figure 1

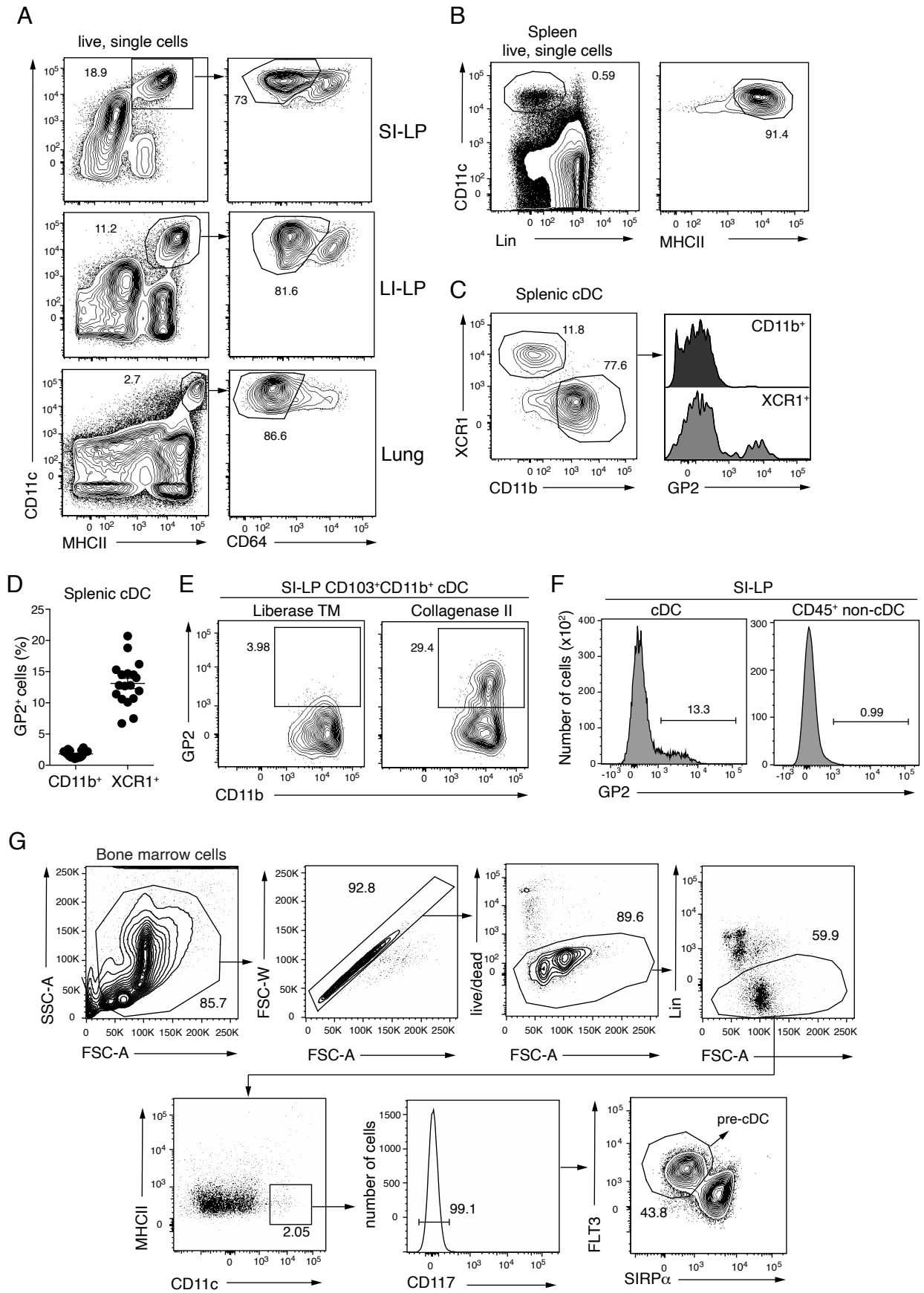

Supplement: Supplementary file 1 — Figure S1. [file SJI-96-e13219-s004.pdf]

## Supplementary Figure 2

A

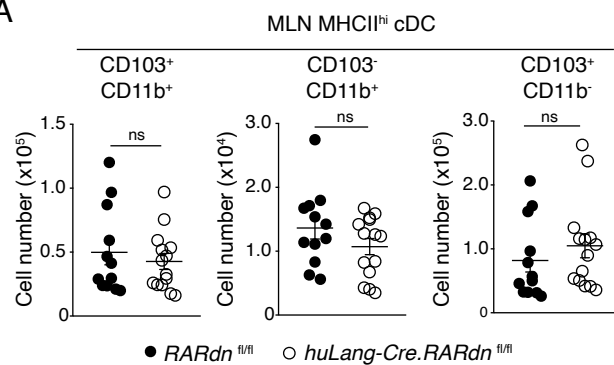

B

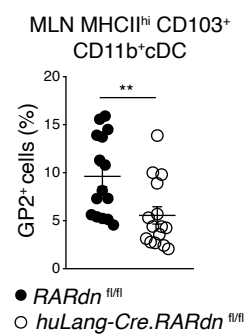

Supplement: Supplementary file 2 — Figure S2. [file SJI-96-e13219-s001.pdf]

Supplementary Figure 3

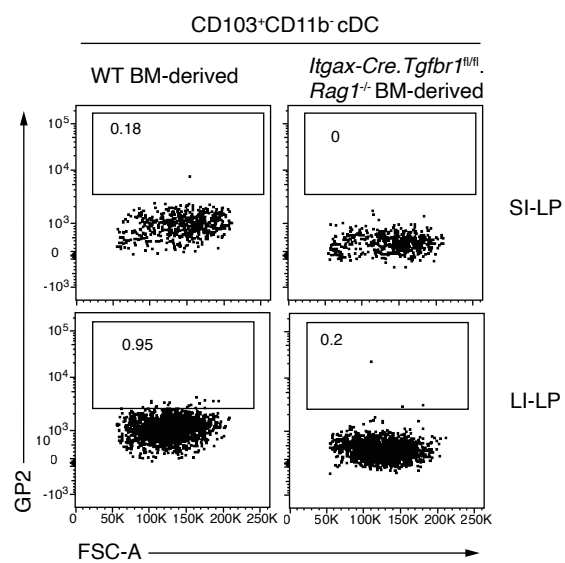

Supplement: Supplementary file 3 — Figure S3. [file SJI-96-e13219-s002.pdf]

Supplementary Figure 4

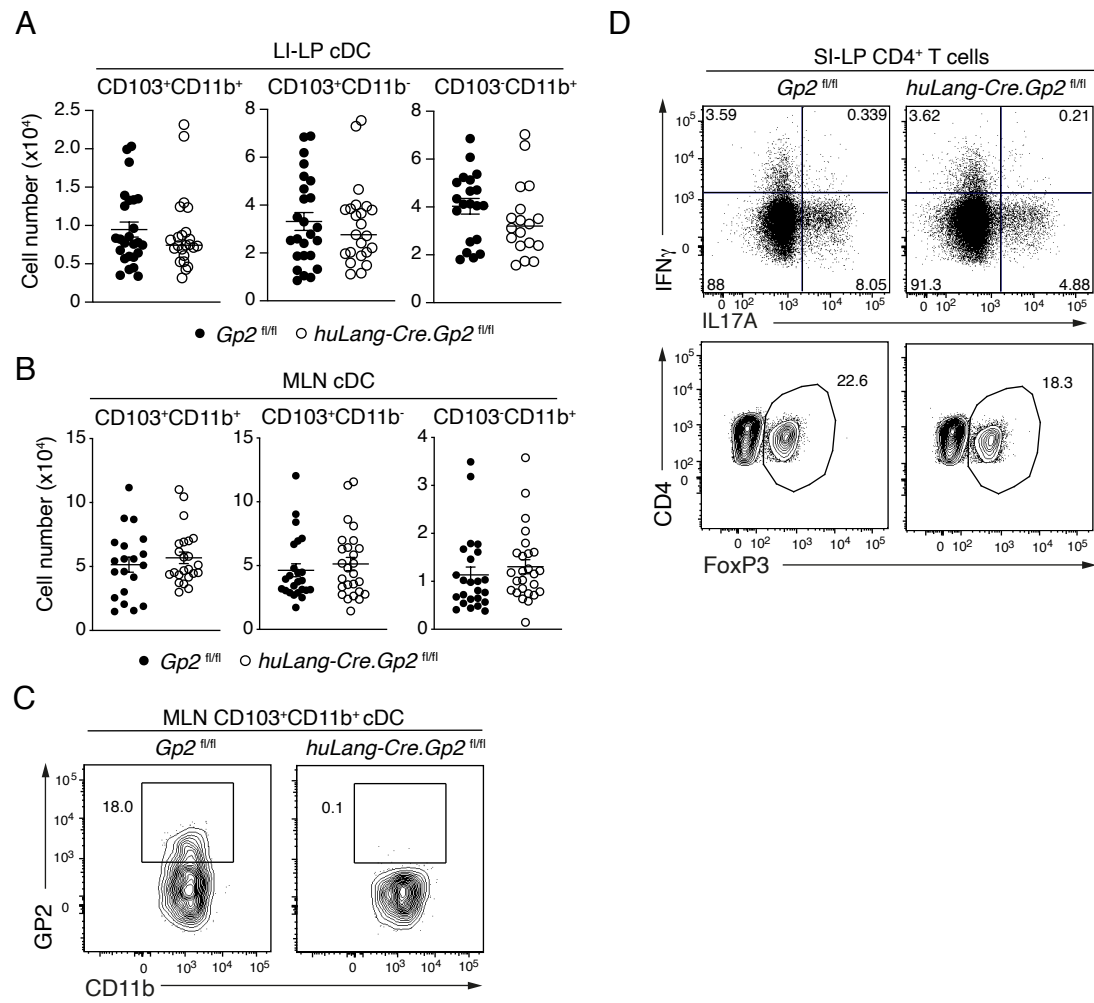

Supplement: Supplementary file 4 — Figure S4. [file SJI-96-e13219-s003.pdf]
